# Extrathyroidal extension is independently associated with cervical lymph node metastasis in pediatric papillary thyroid carcinoma

**DOI:** 10.20945/2359-4292-2026-0088

**Published:** 2026-08-05

**Authors:** Mario Sergio Rocha Macedo, Rodrigo Schuler Honorio, Willer Feitosa Menezes, Paula Assunção Medeiros, Claudio Pinheiro Dias, Clovis Pinheiro Macedo, Luis Alberto Albano Ferreira, Gdayllon Cavalcante Meneses, Wellington Alves Filho

**Affiliations:** 1 Serviço de Cirurgia de Cabeça e Pescoço, Hospital Infantil Albert Sabin, Fortaleza, CE, Brasil; 2 Departamento de Cirurgia/Programa de Pós-Graduação em Ciências Médico-Cirúrgicas, Universidade Federal do Ceará, Fortaleza, CE, Brasil; 3 Departamento de Patologia, Hospital Infantil Albert Sabin, Fortaleza, CE, Brasil; 4 Programa de Residência em Cirurgia Pediátrica, Hospital Infantil Albert Sabin, Fortaleza, CE, Brasil; 5 Departamento de Cirurgia Pediátrica, Hospital Infantil Albert Sabin, Fortaleza, CE, Brasil; 6 Departamento de Análises Clínicas e Toxicológicas, Faculdade de Farmácia, Universidade Federal do Ceará, Fortaleza, CE, Brasil

**Keywords:** Pediatric thyroid cancer, papillary thyroid carcinoma, extrathyroidal extension, cervical lymph node metastasis, risk stratification

## Abstract

**Objective:**

To identify clinicopathologic predictors independently associated with cervical lymph node metastasis in pediatric papillary thyroid carcinoma.

**Subjects and methods:**

We performed a retrospective cohort including patients younger than 18 years who underwent total thyroidectomy for papillary thyroid carcinoma (PTC) (2013-2023) at a tertiary pediatric hospital in Brazil. Clinical and histopathologic variables were reviewed per World Health Organisation Classification 5th edition (2022) and the College of American Pathologists protocol (2023). Multivariable associations with cervical lymph node metastasis (LNM) were estimated using Firth’s penalized logistic regression; discrimination was summarized by the area under the ROC curve (AUC) with 95% bootstrap confidence intervals.

**Results:**

Thirty-two patients were included (median age, 14 years; 81% female). Cervical LNM occurred in 78%. Extrathyroidal extension (ETE) was associated with cervical lymph node metastasis in bivariate analysis (100% vs 59%; p = 0.007) and remained independently associated with cervical lymph node metastasis after adjustment for tumor T stage and lymphatic invasion (OR, 14.29; 95% CI, 1.28-2028.82; p = 0.028).

**Conclusion:**

ETE was independently associated with cervical lymph node metastasis in pediatric PTC and may justify closer postoperative surveillance.

## INTRODUCTION

Although uncommon, papillary thyroid carcinoma (PTC) in children and adolescents has shown a rising incidence over recent decades, particularly among adolescent girls. This increase may reflect, at least in part, enhanced diagnostic surveillance with widespread use of imaging and biopsy, together with environmental and biological determinants that continue to be elucidated (^1^). While pediatric PTC shares features with adult disease, it exhibits distinctive clinical and biological behavior, including a higher frequency of extrathyroidal extension, elevated rates of cervical lymph node metastasis, and, in some cases, pulmonary metastasis at diagnosis (^2^).

Cervical lymph node metastases are present in approximately 70% of children with PTC (^3^). Although the impact on overall survival is limited, nodal involvement is a well-established prognostic factor for locoregional recurrence and directly influences follow-up strategy, the target level of TSH suppression, and the extent of surgery (^2^,^4^). Multiple clinicopathologic variables have been proposed as predictors of cervical nodal disease, including age, sex, tumor size, multifocality, extrathyroidal extension (ETE), capsular infiltration, positive surgical margins, and aggressive histologic variants such as the diffuse sclerosing subtype (^5^-^7^). However, published results are heterogeneous, partly due to small sample sizes and methodological differences across studies.

Within this context, individualized follow-up becomes particularly relevant. A proportion of patients evolve with indeterminate or biochemically incomplete response after initial therapy. According to the 2025 American Thyroid Association (ATA) management guidelines for adult differentiated thyroid cancer, such scenarios may reflect previously unrecognized cervical lymph-node metastases and often correspond to persistent or recurrent nodal disease (^8^). Similar risk-adapted principles have been increasingly considered in pediatric differentiated thyroid cancer because of its high initial nodal burden and excellent survival (^2^). Identifying, at baseline, the factors associated with cervical lymph node involvement can therefore refine risk stratification, guide individualized surveillance, and optimize clinical decision making. Hence, the purpose of this study is to identify independent predictors of cervical lymph node metastasis in pediatric PTC.

## SUBJECTS AND METHODS

We conducted a retrospective cohort study based on medical record review of patients who underwent total thyroidectomy for PTC at a tertiary pediatric hospital in Northeastern Brazil between January 2013 and May 2023. The study was approved by the institutional Research Ethics Committee (CAAE 70588423.8.0000.5042; approval No. 6.299.762) and was conducted in accordance with the Declaration of Helsinki (2013 revision).

During the study period, 36 patients younger than eighteen years were evaluated. Thirty-two patients with PTC were included in the analysis, with 4 exclusions for not meeting the study scope. All histologic slides were reviewed by a single pathologist according to the 5th edition of the World Health Organization (WHO) Classification of Tumors (2022) and the College of American Pathologists (CAP) protocol, version 4.4.0 (2023) (^9^,^10^).

We collected clinical variables (age, sex) and histopathologic features (subtype, multifocality, extrathyroidal extension, lymphatic invasion, perineural invasion, surgical margins, mitotic index, and TNM staging per AJCC 8th edition). Microscopic extrathyroidal extension was defined as tumor extension into the strap muscles or perithyroidal soft tissues identified on histopathologic examination, without gross or intraoperative evidence of macroscopic invasion, in accordance with the College of American Pathologists protocol and the WHO Classification of Tumors. We also recorded the ATA 2015 recurrence-risk category and use of radioiodine therapy. The primary endpoint was the presence of cervical lymph node metastasis in the central and/or lateral compartments. A case was classified as positive if cervical lymph node metastasis was histologically confirmed either at the time of the initial thyroidectomy or neck dissection, or during any subsequent lymph node surgery performed for persistent or recurrent disease. For analysis, the primary outcome was categorized as metastasis present, defined as any histologically proven cervical nodal metastasis from diagnosis to last follow-up, or metastasis absent. Neck dissections were not performed prophylactically in this cohort. Lateral neck dissections (levels II-V) were undertaken only when lymph node metastasis was documented preoperatively by cytology consistent with papillary thyroid carcinoma. Central compartment dissections (level VI) were performed when there was clinical, imaging, cytologic, or intraoperative suspicion of nodal involvement. Follow-up time was counted from diagnosis to last contact or transition to adult care on reaching eighteen years of age.

### Statistical analysis

Statistical analyses were performed using GraphPad Prism 8.0 (GraphPad Software, USA) and R version 4.5.0 (R Foundation, Austria). Categorical variables were summarized as counts and percentages. Continuous variables were tested for normality using the Shapiro-Wilk test and, due to nonparametric distribution, were reported as median and interquartile range. Bivariate comparisons were conducted using Fisher’s exact test or the Mann-Whitney test, as appropriate. Variables with univariable *p* < 0.20 were entered into the multivariable model. Multivariable analysis was performed using Firth-penalized logistic regression, selected to account for small sample size, sparse categories, and potential quasi-separation. The model included three variables for 25 events, approximately eight events per variable. Odds ratios (ORs) with 95% confidence intervals (CIs) were derived from Firth’s method. Model discrimination was evaluated using the area under the receiver operating characteristic (ROC) curve (AUC) based on predicted probabilities, with 95% CIs obtained by stratified bootstrap with 2,000 resamples. The optimal cutoff was defined by Youden’s index, and corresponding sensitivity and specificity were reported. As a sensitivity analysis, AUC was also estimated using DeLong’s method with concordant results; bootstrap CIs are reported.

## RESULTS

**Figure 1:**
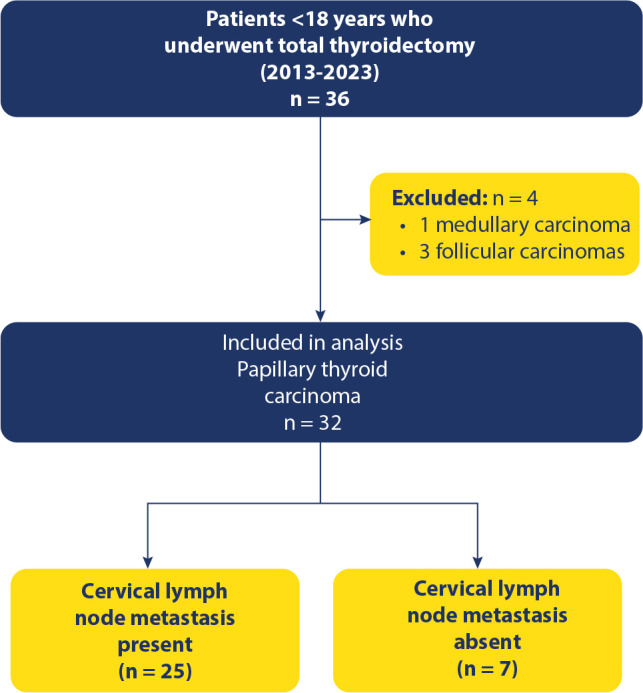
Study flow diagram of cohort selection and classification by cervical lymph node metastasis.

A total of 32 pediatric patients with papillary thyroid carcinoma who underwent total thyroidectomy were included (**Figure 1**). The median age was 14 years [IQR, 10-15.7], ranging from 4 to 17 years, with a predominance of females (81%). Most tumors corresponded to the classic variant, followed by the encapsulated classic, diffuse sclerosing, solid/trabecular, infiltrative follicular, and oncocytic subtypes. Multifocality was present in 37.5% of cases, extrathyroidal extension in 47%, and lymphatic invasion in 59%. Mitotic activity was uniformly low across the cohort, and only one patient exhibited tumor necrosis on histologic review. Upon reassessment, this case showed combined morphologic features consistent with high-grade transformation and was reclassified as high-grade papillary thyroid carcinoma. Regarding T staging, T1b tumors predominated. Cervical lymph node metastasis confirmed by histopathology was observed in 78% of patients during the disease course, including cases identified at the initial surgery and during subsequent procedures throughout follow-up. According to the 2015 ATA risk stratification, 34% were classified as low risk, 38% as intermediate risk, and 28% as high risk. Half of the patients (16/32) received adjuvant radioiodine therapy, and three (9.3%) had distant pulmonary metastases identified on whole-body radionuclide scans during follow-up. The median follow-up was 28 months [IQR, 17-44], and one patient was lost to follow-up.

In the bivariate analysis, age showed no association with the presence of lymph node metastasis, either as a continuous variable (median age 13 years in patients with metastasis vs 15 years in those without; p = 0.20) or when categorized as ≤12 years and >12 years (p > 0.999). Sex was also not significant (p = 0.29), although all six male patients presented cervical metastasis. Multifocality was not associated with metastasis (p = 0.68). Likewise, positive surgical margins (p = 0.30) and perineural invasion (p > 0.999) showed no significant relationship with cervical nodal metastasis. By contrast, extrathyroidal extension (100% vs 59%; p = 0.007), lymphatic invasion (95% vs 54%; p = 0.010), and advanced tumor stage (T1b-T3a: 91% vs T1a: 44%; p = 0.010) were associated with lymph node involvement (**Table 1**).

**Table 1:** Clinicopathologic features and bivariate analysis by cervical lymph node metastasis in 32 pediatric patients with papillary thyroid carcinoma

Variable	Total (N = 32)^*^	Cervical lymph node metastasis		*p* value^**^
		Absent (n = 7)	Present (n = 25)	
Age (years)	14.0 [10.0-15.5]	15.0 [13.5-16.0]	13.0 [9.0-15.0]	0.20
Sex				0.29
Female	26 (81%)	7	19	
Male	6 (19%)	0	6	
Pathological subtype				-
Classic	19 (59.3)	5	14	
Encapsulated classic	3 (9.4)	2	1	
Diffuse sclerosing	4 (12.5)	0	4	
Infiltrative follicular	1 (3.1)	0	1	
Oncocytic	1 (3.1)	0	1	
Solid/trabecular	3 (9.4)	0	3	
High-grade papillary carcinoma	1 (3.1)	0	1	
Multifocality				0.68
Absent	20 (62.5)	5	15	
Present	12 (37.5)	2	10	
Margins				0.30
Free	25 (78.1)	7	18	
Involved	7 (21.9)	0	7	
Perineural invasion				> 0.999
Absent	30 (93.8)	7	23	
Present	2 (6.2)	0	2	
Extrathyroidal extension				0.007
Absent	17 (53.1)	7	10	
Present	15 (46.9)	0	15	
Lymphatic invasion				0.010
Absent	13 (40.6)	6	7	
Present	19 (59.4)	1	18	
Vascular invasion				-
Absent	32 (100)	7	25	
Mitotic index^†^				-
0	20 (62.5)	4	16	
1	6 (18.8)	1	5	
2	5 (15.6)	2	3	
3	1 (3.1)	0	1	
Necrosis				-
No	31 (97%)	7	24	
Yes	1 (3.1%)	0	1	
T stage(AJCC)^††^				0.010
T1a	9 (28.1)	5	4	
T1b	12 (37.5)	1	11	
T2	9 (28.1)	1	8	
T3a	2 (6.2)	0	2	
N Stage				
N0	7 (^22^)	-	-	-
N1a	14 (44)	-	-	
N1b	11 (34)	-	-	
Cervical metastasis				-
Absent	7 (21.9)	-	-	
Present	25 (78.1)	-	-	
Distant metastasis				-
Absent	29 (90.7)	-	-	
Present	3 (9.3)	-	-	
Recurrence risk (ATA)				-
Low	11 (34.4%)	-	-	
Intermediate	12 (37.5%)	-	-	
High	9 (28.1%)	-	-	
Radioiodine therapy				-
Yes	16 (50%)			
No	16 (50%)			

* Categorical variables are reported as n (%); continuous variables as median [IQR]

** p values were obtained using Fisher’s exact test for categorical variables and Mann-Whitney for continuous variables; two-sided p < 0.05 was considered statistically significant.

† Mitotic index is reported as mitoses per 2 mm^2^ (WHO 2022).

†† T stage comparison: T1a vs T1b, T2, T3a (AJCC 8th edition).

**Figure 2:**
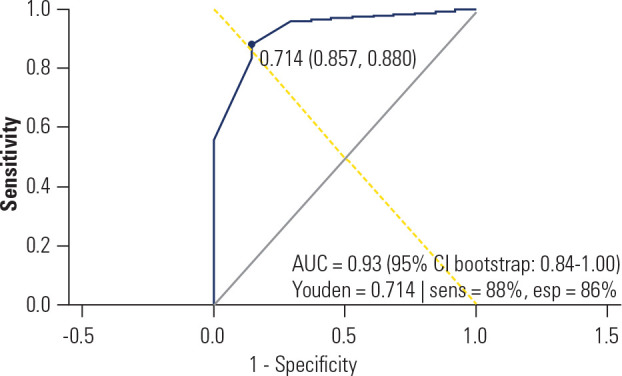
ROC curve for the multivariable Firth’s penalized logistic regression model predicting cervical lymph node metastasis (variables included in the model: T stage, extrathyroidal extension, lymphatic invasion). The optimal cutoff by Youden’s index was 0.714 (sensitivity 88%, specificity 86%). AUC = 0.93; 95% CI by bootstrap, 0.84-1.00.

After adjustment for covariates in a Firth-penalized logistic model, extrathyroidal extension remained independently associated with cervical lymph node metastasis (OR, 14.29; 95% CI, 1.28-2028.82; p = 0.028). Tumor stage showed a trend toward association (OR, 5.95; 95% CI, 0.49-119.98; p = 0.162), whereas lymphatic invasion did not reach statistical significance (OR, 2.97; 95% CI, 0.20-49.61; p = 0.407) (**Table 2**). The overall model was statistically significant (likelihood-ratio test: χ^2^ = 14.30; df = 3; p = 0.0025). The ROC curve yielded an AUC of 0.93 (95% CI by bootstrap, 0.84-1.00); Youden’s index was 0.714, with sensitivity of 88% and specificity of 86%. The positive predictive value (PPV) and negative predictive value (NPV) at the optimal cutoff were 0.96 and 0.67, respectively (**Figure 2**).

**Table 2:** Firth’s penalized logistic regression for clinicopathologic predictors of cervical lymph node metastasis in children and adolescents with papillary thyroid carcinoma

Variable	OR (95% CI)	p value^*^
Tumor stage (T)	5.95 (0.49 - 119.98)	0.162
Extrathyroidal extension	14.29 (1.28 - 2028.82)	0.028†
Lymphatic invasion	2.96 (0.20 - 49.61)	0.407

* Two-sided p < 0.05 was considered statistically significant

† Statistically significant.

OR: odds ratio; CI: confidence interval. Model fitted using Firth’s penalized logistic regression.

## DISCUSSION

In this single-center cohort of children and adolescents with papillary thyroid carcinoma, we observed a high burden of cervical lymph node metastasis and found that extrathyroidal extension was the only factor independently associated with nodal involvement after multivariable adjustment. The predictive model showed strong discrimination, supporting the use of extrathyroidal extension to refine risk stratification, inform surgical planning, and guide closer postoperative surveillance.

The increasingly widespread use of ultrasonography in clinical practice, together with a growing concern about thyroid cancer, has contributed to the rising diagnosis of thyroid nodules and PTC in children and adolescents, particularly among females. Although the overall prognosis is excellent, the disease imposes a meaningful burden on patients and families because it entails a chronic oncologic condition requiring long-term follow-up (^11^).

In our cohort, the age at diagnosis and the female predominance aligned with international pediatric series (^12^). The classic variant was most frequent, followed by the encapsulated classic variant, whereas the diffuse sclerosing variant, although less prevalent, deserves attention due to its association with more aggressive behavior reported in several studies (^13^,^14^).

Multifocality, extrathyroidal extension, and lymphatic invasion occurred at rates comparable to those described in other pediatric series (^5^,^6^). In accordance with the 5th edition of the WHO Classification (2022) and the CAP protocol (v4.4.0.0), vascular and lymphatic invasion were reported independently. No vascular invasion was identified, while lymphatic invasion was commonly observed, reinforcing the propensity of PTC to spread via lymphatic pathways (^9^,^10^). Cervical lymph node metastases were frequent during the disease course and fell within the range reported by pediatric cohorts (^5^,^6^). Pulmonary metastases were uncommon and within the frequency described in larger studies (^10^). Adjuvant radioiodine was used in about half of the cohort, guided primarily by stimulated thyroglobulin, cervical ultrasound, and post-therapy whole-body scans rather than strictly by ATA risk category (^2^,^6^).

We evaluated clinicopathologic factors associated with cervical lymph node metastasis, including both the central and lateral compartments, considering findings at initial diagnosis and throughout the disease course. Age was not associated with metastasis, either as a continuous variable (*p* = 0.20) or when categorized as ≤12 vs >12 years (*p* > 0.99), in accordance with the 2015 Pediatric ATA guideline recommendation to distinguish prepubertal and postpubertal periods (^2^). This result contrasts with previous series reporting greater aggressiveness and higher nodal burden in prepubertal children compared with adolescents (^15^,^16^). Similarly, sex was not associated with metastasis (*p* = 0.29), although all male patients presented lymph node involvement.

The diffuse sclerosing variant of papillary thyroid carcinoma accounted for 4 of 32 cases (12.5%). All presented with cervical metastasis at diagnosis; across the cohort, three patients had pulmonary metastases, including one within this subtype. Using the classic and encapsulated classic variants as the less aggressive reference group, the diffuse sclerosing subtype showed a higher frequency of cervical nodal involvement, although the difference was not statistically significant. Larger pediatric series have reported higher incidences of central and lateral nodal disease and a greater likelihood of pulmonary metastasis with this variant (^14^,^17^). Differences in study design, such as systematic dissection of central and lateral levels, as well as the small sample size, may account for discrepancies. Multifocality, positive surgical margins, and perineural invasion were not associated with cervical metastasis in our cohort. By contrast, extrathyroidal extension and lymphatic invasion were associated with nodal disease, and a more advanced tumor stage (T1b-T3a vs T1a) also showed association in bivariate analyses. These findings are consistent with pediatric literature identifying extrathyroidal extension, lymphatic invasion, and tumor size/T stage as the main predictors of cervical metastasis, whereas multifocality, margins, and perineural invasion yield inconsistent results (^5^,^6^,^18^,^19^). We did not analyze necrosis or mitotic index statistically because mitotic counts were uniformly low; the single tumor with necrosis was the case reclassified as high-grade papillary thyroid carcinoma according to WHO 2022, precluding meaningful comparisons (^20^).

Extrathyroidal extension plays a key role in the locoregional biology of papillary thyroid carcinoma in children and is the only independent predictor of nodal involvement with cervical lymph node metastasis in the central and lateral compartments (^21^-^23^). In our cohort, when present, extrathyroidal extension was microscopic. Although the AJCC 8th edition restricts T staging to gross invasion, even minimal microscopic extension may be biologically relevant in pediatrics (^22^,^24^). After adjustment for covariates in a Firth-penalized logistic model, ETE remained independently associated with nodal involvement, whereas tumor stage showed only a trend and lymphatic invasion did not retain significance. The model’s ROC analysis demonstrated strong discriminative ability; however, given the single-center design and limited sample size, this performance should be interpreted as internally validated, and external validation in independent pediatric cohorts is warranted before broader clinical application. Taken together, these findings support using extrathyroidal extension to refine risk stratification and to plan closer postoperative cervical surveillance.

This study has some limitations. It was retrospective and conducted at a single tertiary center, which may limit generalizability, and the sample size was relatively small, reflecting the lower frequency of papillary thyroid carcinoma in the pediatric population. The number of outcome events was also limited, so some estimates should be interpreted with caution. Furthermore, near-complete separation was observed when conventional logistic regression was initially applied, which led us to adopt Firth’s penalized logistic regression to obtain more stable estimates. In addition, although neck dissections were not performed prophylactically, the indication for central compartment dissection was not always based on preoperative cytologic confirmation and in some cases relied on intraoperative suspicion of nodal involvement, which may have introduced a degree of selection bias and increased the likelihood of detecting cervical lymph node metastases in this cohort. Histopathologic evaluation was performed by a single pathologist, which does not allow assessment of interobserver variability, and the absence of independent blinded review by multiple pathologists represents an additional limitation. Nonetheless, the study has important strengths: histologic slide evaluation followed the most recent WHO and CAP criteria, ensuring diagnostic consistency; standardized ATA pediatric risk stratification and AJCC 8th-edition staging were applied; and Firth’s penalized logistic regression was used to reduce small-sample bias.

In conclusion, extrathyroidal extension was the only factor independently associated with cervical lymph node metastasis in children and adolescents with papillary thyroid carcinoma. These data support considering ETE as an informative variable within dynamic risk stratification to help individualize postoperative surveillance; however, prospective multicenter studies are needed to determine how best to integrate ETE into pediatric care.

## Data Availability

datasets related to this article will be available upon request to the corresponding author.
